# Oral, Cloacal, and Hemipenal Actinomycosis in Captive Ball Pythons *(Python regius)*

**DOI:** 10.3389/fvets.2020.594600

**Published:** 2021-01-08

**Authors:** Steven B. Tillis, Marley E. Iredale, April L. Childress, Erin A. Graham, James F. X. Wellehan, Ramiro Isaza, Robert J. Ossiboff

**Affiliations:** ^1^Department of Comparative, Diagnostic, and Population Medicine, College of Veterinary Medicine, University of Florida, Gainesville, FL, United States; ^2^Department of Large Animal Clinical Sciences, College of Veterinary Medicine, University of Florida, Gainesville, FL, United States

**Keywords:** actinomycosis, cloacitis, granuloma, hemipenis, reproductive disease, reptile, sexually transmitted disease, snake

## Abstract

Ball pythons (*Python regius*) are one of the most commonly kept and bred reptiles in captivity. In a large ball python breeding colony, a unique syndrome characterized by granulomatous inflammation of the cloaca and hemipenes (phalli) was observed in 140 of 481 (29.1%) breeding males, but only one of 1,446 breeding females. Lesions were absent in virgin males (*n* = 201) and virgin females (*n* = 293). On postmortem examination (*n* = 13, 12 males, 1 female), numerous well-defined mucosal and submucosal granulomas were present in the hemipenes (males) and cloaca (males and female). Extension into the coelomic cavity and liver was noted in a subset of these animals. An additional small subset of breeder animals (6/2027; 0.3%) presented with oral and mandibular swellings. Postmortem examination (*n* = 4, all female) showed oral lesions histologically indistinguishable from the cloacal/hemipenal lesions. Aerobic bacterial culture of a hepatic granuloma of one snake resulted in the isolation of filamentous, Gram-positive bacilli; amplification, and sequencing of the 16S rRNA gene and subsequent phylogenetic analysis of the isolate identified the bacterium as a novel species of *Actinomyces*. Screening of cloacal and oral granulomas using a specific, heminested 16S rRNA PCR assay confirmed the presence of the agent in all 17 snakes, as well as in cloacal swabs taken at the time of necropsy in 11/13 snakes. The *Actinomyces* sp. was also identified by PCR of cloacal swabs of unaffected snakes (*n* = 94) from the affected colony and two unrelated, grossly unaffected breeding colonies. In the affected colony, 65.5% of breeding animals (*n* = 23) but only 11.9% of virgin animals (*n* = 42) tested PCR positive, with breeding status being a significant predictor of bacterium presence (*P* < 0.00001). This study characterizes a granulomatous mucosal disease syndrome of breeding male ball pythons associated with a novel *Actinomyces*. In stark contrast to male snakes, the presence of the bacterium in both breeding and virgin females was very rarely associated with clinical disease. Though additional studies are necessary, these data suggest a role for the novel bacterium in the disease process, a predilection for clinical disease in male snakes, and the potential for sexual transmission of the disease.

## Introduction

The captive reptile trade is a large and diverse market within the United States. Over 4.7 million households hold 13.6 million pet reptiles, comprising a market with annual revenues of over a billion dollars (1). Among captive reptiles, ball pythons *(Python regius)* have become one of the most popular pet reptiles kept and bred in captivity due to their small size, mild disposition, hardy nature, and variety of colors (2). Large colonies of these pythons numbering in the hundreds to thousands are kept and bred to supply pet trade demands. Currently over 6,000 color combinations are listed, with hundreds of unique genetic mutations combined to create new and unique phenotypes (3). Shifts in market demands for various color phenotypes require a breeding colony to keep significant numbers of snakes with diverse genetic backgrounds to produce offspring with the highest market demand annually.

In captive snake breeding colonies, several important reproductive diseases can occur. Reproductive diseases common in breeding females are often sequela of dystocia and include ovostasis (egg binding), oviductal prolapse, and egg-yolk coelomitis. In breeding males, common reproductive pathology includes hemipenal prolapse and hemipenal trauma, potentially caused by strenuous reproductive activity and aggressive copulation. Cloacitis associated with reproduction and associated courtship and copulation can be a major disease in both sexes (4).

Currently, no sexually transmitted diseases of a bacterial or viral nature in non-avian reptiles are formally described in the literature. Although sexual transmission of nematodes has been documented in wild *Anolis* lizards (5), there is additional concern for the potential sexual transmission of various reptile pathogens, including protozoa, bacteria, and other potential pathogens (4). However, bacterial venereal diseases are well-documented in birds (6). Among poultry species, a variety of sexually transmitted bacteria have been described including *Mycoplasma meleagridis* in turkeys, *M. iowae* in chickens, and *M. cloacale* in domestic geese (6). Moreover, contamination of chicken and turkey semen with *Salmonella* and *Campylobacter* spp. can contribute to the spread of these agents in both artificial and traditional breeding methods (7–9).

Members of the phylum Actinobacteria are often filamentous and fastidious and are relatively poorly identified by standard biochemical methods. The phylum contains some highly significant animal pathogens, including the genera *Mycobacterium* and *Nocardia*. *Actinomyces* is a bacterial genus containing at least 45 species in the phylum Actinobacteria, order Actinomycetales, family Actinomycetaceae. Members of the *Actinomyces* are common inhabitants of oral and genital mucosa in animal species, including humans (7, 10, 11). Furthermore, members of Actinobacteria have been identified both in the cloaca of birds (12) and in the gut microbiomes of various agamids (13). While they are most commonly innocuous members of the mucosal microbiome, certain species are also causative agents of inflammatory processes (actinomycoses) of the oral cavity, reproductive tract, abdomen, and subcutis (7, 14). Still other Actinobacteria such as those in the genus *Devriesea* have been shown to be directly involved with the formation of dermal lesions in agamid lizards (15).

This study identifies and characterizes a novel *Actinomyces* species associated with a granulomatous disease syndrome of oral, cloacal, and hemipenal tissues in captive ball pythons by epidemiological, pathological, and molecular modalities.

## Materials and Methods

### Snake Population and Clinical Investigation

The investigated population is a large commercial production colony of ball pythons (*Python regius*) consisting mostly of animals with captive-bred origins. The colony consists of ~2,000 breeding animals and produces neonates to supply the pet trade. Facility health records and animal information are maintained in a Microsoft Access database. In this colony, a breeding group consists of 2 males assigned to breed 6 females each year. Starting in February, males are introduced to females until either the female ovulates or the season ends in August. Each male is separately introduced to one female at a time and is moved to the next female in the breeding group 3 times a week, spending at least 2 days with each female. In this way, each of the two males is exposed to each female in the breeding group at least once every 2 weeks during the breeding season.

Anecdotal recollection dates the first recognition of the disease syndrome of male ball pythons (initially noted by the producer as abnormal cloacal irritation) a decade or more prior to this study. At that time, disease status determination was limited to observations of sporadic cases of occult irritation on the external perimeter of the cloaca in a small portion of breeding males. In attempts to potentially remedy the irritation, the practice of manually everting breeding males' hemipenes was adopted in the facility to check for any adhered substrate. Through this practice, a spectrum of gross syndrome expression was noted to include irritation and small masses on the hemipenes, in addition to the irritation previously noted around the cloaca.

To determine a farm-level prevalence of the disease syndrome, a complete census of all 481 breeding males and 1,446 breeding females on the farm was performed in September 2017. The cloacae and manually extruded hemipenes (males only) of all breeding snakes were examined for presence of any masses. Data recorded for the census included gross disease presence (present/absent), sex (male/female), and age (years). The association between disease presence and sex was evaluated with a Chi-squared analysis, with significance defined as *p* < 0.05. All statistical calculations for this study were performed with R Studio.

In January 2018, in preparation for the breeding season, the producer removed 168 breeding males from the breeding colony, which included 87 animals identified in the 2017 census with the disease syndrome. The lone female identified with the disease syndrome in 2017 was also removed from the colony. One hundred and sixty six first-time breeding males were added to breeding groups for the 2018 season. For the 2018 season, the producer placed 388 animals with no previous signs of the disease syndrome in breeding groups with partnering males also without signs, and 38 males with no previous signs in breeding groups with a partnering male that had signs of the disease syndrome in 2017.

To examine incidence of new cases as well as determine an updated prevalence, a second census was conducted in October 2018. This census included all 479 breeding males and 1,477 breeding females, as well as 201 virgin males and 293 virgin females. Data recorded for this census included 2018 gross disease presence (present/absent), 2017 gross disease presence (present/absent/not applicable), sex (male/female), age (years), breeding status (breeder/virgin), and, for breeding males, breeding partner gross disease presence (present/absent). The association between gross disease presence and sex, gross disease presence and breeding status, and gross disease presence and breeding partner gross disease presence was evaluated with a Chi-squared analysis, with significance defined as *p* < 0.05. Finally, in September 2019 a smaller subset of animals in the colony were identified with swelling in the oral or nasal cavity. A third and final census was performed on all 2,027 breeding animals in the facility to determine the prevalence of facial swelling.

For molecular diagnostic investigation on a possible etiology, cloacal swabs were taken from 10 breeding female ball pythons, 12 virgin females, and 30 virgin males as well as 12 males and one female with the disease syndrome. The 10 breeding females had known exposure with a male with the disease syndrome and were selected using stratified pseudorandom sampling wherein a facility employee picked an animal from each cluster of caging organized by genetic morphological phenotype. Virgin animals in the affected colony were chosen by generating a list of applicable animals and assigning each a sequential numerical digit with animals selected at random, using https://www.randomizer.org. At the time of swabbing, disease status was confirmed as present or absent via gross examination of the cloaca and hemipenes. Variables collected for analysis include cloacal swab PCR bacterial presence (present/absent), gross disease presence (present/absent), sex (male/female), breeding status (breeder/virgin), and source colony status (colony absent of disease syndrome/disease syndrome present in colony). The association between cloacal bacterial presence and sex and cloacal bacterial presence and breeding status was determined with a Chi-squared analysis with a significance of 0.05. Additionally, an unweighted Cohen's Kappa test was performed to determine the level of agreement between cloacal bacterial presence and lesion presence. For males, the hemipenes were manually extruded, and the swab was run along both hemipenes and anterior and posterior portions of the internal cloacal cavity. For females, swabs were run along the anterior and posterior portions of the internal cloacal cavity. Swabs were placed in sterile microcentrifuge tubes and kept on ice until either frozen (−80°C) or immediately extracted.

Oral swabs were also collected from the affected colony to assess possible oral pathogen prevalence. Oral swabs were taken from two subsets of animals. The first subset was from 48 animals (12 virgin males, 12 virgin females, 12 breeder males, and 12 breeder females) selected at random by assigning each a sequential numerical digit and randomly chosen using https://www.randomizer.org. The second subset of animals included 37 snakes (17 breeder females, 4 breeder males, 8 virgin females, and 8 virgin males) whose cloaca had been sampled previously as part of cloacal swabbing described above. Variables collected for analysis include oral swab PCR bacterial presence (present/absent), sex (male/female), breeding status (breeder/virgin), and cloacal swab PCR bacterial presence (present/absent) for the second subset of animals. The association between oral bacterial presence and cloacal bacterial presence was determined with a Fisher's exact test with a significance of 0.05. Oral swabs were collected by running a Rayon-tipped plastic shaft swab along the oral mucosa at the labial margin, the trachea, the choana, and the caudal oral cavity and cranial esophagus. Swabs were placed in sterile microcentrifuge tubes and kept on ice until frozen (−80°C) or were immediately extracted.

Also included in this report are 2 smaller colonies consisting of ~40 ball pythons each, with all animals of captive bred origins. These colonies have no history of the disease syndrome and use a breeding strategy similar to that outlined above but differ in having only 1 male in a given breeding rotation. For the purposes of this study, animals in all 3 colonies were classified into 4 groups: breeder males and females, and virgin males and females. For comparison and screening purposes, cloacal swabs were also taken from the two smaller colonies of ball pythons with no history of the disease syndrome with a combined total of 10 breeding males, 12 breeding females, 10 virgin males, and 10 virgin females. For the smaller colonies with no history of cloacal disease, nearly all demographically comparable animals were sampled to supply similar sample sizes.

### Postmortem Examinations

Complete postmortem examinations were performed on 17 adult breeding animals with grossly apparent lesions; 13 snakes (12 males; 1 female) had cloacal/hemipenal lesions and four snakes (4 females) had oral lesions. All snakes were chemically euthanized by the Zoological Medicine Service at the University of Florida Small Animal Hospital for quality of life and/or population health concerns using AVMA approved euthanasia protocols (16). Samples of all tissues were collected and preserved in 10% neutral buffered formalin and submitted for routine processing and sectioning to the Histology Laboratory at the College of Veterinary Medicine, University of Florida. Samples of liver, kidney, heart, lung, spleen, and cloacal and visceral granulomas (when present) were collected and archived frozen (−80°C). In all 17 animals, the cloacal and hemipenal mucosa (males) or cloacal mucosa only (females) were swabbed with a single rayon-tipped plastic shaft swab (Puritan, Guilford, ME); in snakes with oral lesions, the oral mucosa was also swabbed with a separate swab. All swabs were archived frozen (−80°C) until testing. After a minimum of 24 h of fixation, tissues were processed routinely, cut at 5-μm sections, and stained with hematoxylin and eosin. Histochemical stains (Warthin-Starry, Twort's Gram, Brown and Brenn Gram, Giemsa, Gomori methenamine silver, and Fite-Faraco acid fast) were applied to select sections with granulomatous inflammation.

### Bacterial Culture and 16S rRNA PCR

To decrease the chances of culturing a contaminant cloacal bacterium, a single hepatic granuloma was excised using sterile methods and submitted for aerobic bacterial culture to the Clinical Microbiology Laboratory at the College of Veterinary Medicine, University of Florida. Samples from the excised mass were plated for culture on chocolate agar, Columbia blood agar (BAP), MacConkey agar (MAC), and colisitin with nalidixic acid (CNA). All plates were incubated at 25°C.

DNA from an isolated bacterial colony was extracted per manufacturer's recommendation using a Qiagen DNeasy Blood and Tissue Kit (Qiagen, Hilden, Germany). An approximately 1500-bp fragment of the small subunit ribosomal RNA (16S rRNA) gene was amplified by polymerase chain reaction (PCR) as previously described (17). Briefly, 1 μl of 20 μM sense primer 27F (5′-AGA GTT TGA TCM TGG CTC AG-3′) and antisense primer 1492R (5′-GYT ACC TTG TTA CGA CTT-3′) (17) were added to 2 μl of 10 × PCR buffer, 0.8 μl of 50 mM magnesium chloride, 0.4 μl of 10 mM dNTP mixture, 0.1 μl of platinum *Taq* DNA polymerase, 12 μl of molecular grade water, and 3 μl of extracted nucleic acids (Invitrogen, Carlsbad, California). Thermocycler conditions were as follows: 1 cycle at 50°C for 2 min; 1 cycle at 95°C for 10 min, 30 cycles of 95°C for 20 s followed by 50°C for 20 s followed by 72°C for 3 min; and 1 cycle at 72°C for 10 min followed by a hold stage at 4°C. Amplified products were visualized on a 1% agarose gel stained with ethidium bromide. Amplicons of the appropriate size were extracted using a Zymoclean Gel DNA Recovery Kit (Zymo Research, Irvine, California) per manufacturer's recommendation and were submitted for Sanger sequencing to a commercial facility (Genewiz, South Plainfield, New Jersey). Sequences were edited and aligned using Geneious Prime (Auckland, New Zealand). Additional specific sequencing primers were designed and used for commercial sequencing (Genewiz) to get double coverage along the entire length of the amplicon. The resultant sequence was compared to the 16S rRNA sequences of representative species of common bacterial families available through BLASTn.

### Phylogenetic Analysis

The sequence of the novel ball python bacterium was aligned against all 45 characterized species of *Actinomyces*, 5 characterized species of the closely related genus *Trueperella*, and *Mobiluncus mulieris* (Genbank AJ576080) as an outgroup using multiple alignment using fast Fourier transform (MAFFT) (18). Maximum likelihood (gamma distributed rate variation with estimate of proportion of invariable sites with 1,000 bootstrap resamplings) and Bayesian (Mr. Bayes 3.2.6 with gamma distributed rate variation, 4 chains of 2 × 10^6^ generations with 25% burn-in) were performed on the CIPRES server (19–21). Phylogenetic trees were visualized using FigTree software (http://tree.bio.ed.ac.uk/software/figtree/).

### Specific Heminested PCR of Tissue and Swab Samples

DNA from postmortem samples of the cloaca, oral mucosa, and liver containing granulomas was extracted using the Qiagen DNeasy Blood and Tissue Kit (Qiagen, Hilden, Germany). Approximately 100 mg of affected tissue was lysed at 56°C for 16 h with proteinase K, and extractions were conducted according to the manufacturer's tissue protocol. DNA extraction from all swabs was performed using Prepman Ultra solution (Applied Biosystems, Foster City, California, US) using modified product protocols. One hundred fifty microliter of Prepman Ultra solution was added to the microcentrifuge tube with the swab and vortexed for 60 s. The tubes were then placed on a 95°C hotplate for 10 min. Once cooled to room temperature, a 1:10 dilution was made using 10 μl of Prepman and sample solution combined with 90 μl of molecular grade water for a final extraction volume of 100 μl.

A specific heminested PCR was developed for detection of the novel *Actinomyces* sp. The determined 16S sequence was compared to sequences of other *Actinomyces* and closely related genera present in Genbank (https://www.ncbi.nlm.nih.gov/genbank/) to develop novel *Actinomyces*-specific primers in the assay. The first round of primers in the protocol are sense primer ActBP154F (5′-GGA TAT TCT GTC TCC TGC GCA-3′) and antisense primer ActBP836R (5′-CTA CGG CGC GGA AAC CAT-3′). The second round in the heminested protocol utilizes the sense primer from the first round (ActBP154F) and antisense primer ActBP614R (5′-TAC CCA CCG CAA GCT GAA AG-3′). PCR reactions were performed with 2X PCRBIO HS Taq Mix kit (PCR Biosystems, Wayne, Pennsylvania) with 2 μl 10 μM of each primer, 25 μl of 2 × PCRBIO HS Taq Mix solution, 18 μl of molecular grade water, and 3 μl of DNA extract (round one) or PCR product (round two). Cycle conditions for both rounds are as follows: 1 cycle at 95°C for 2 min, 40 cycles of 95°C for 20 s, annealing at 57°C for 20 s, and elongation at 72°C for 3 min; 1 cycle at 72°C for 10 min and a hold at 4°C. PCR products were run on a 1% agarose gel. DNA was extracted from excised bands with Zymoclean Gel DNA Recovery Kit (Zymo Research, Irvine, California) per manufacturer's recommendation. All extracted products were submitted for Sanger sequencing (Genewiz). Sequences were edited and aligned using Geneious Prime (Auckland, New Zealand).

## Results

### Snake Population and Clinical Investigation

**Figure 1:**
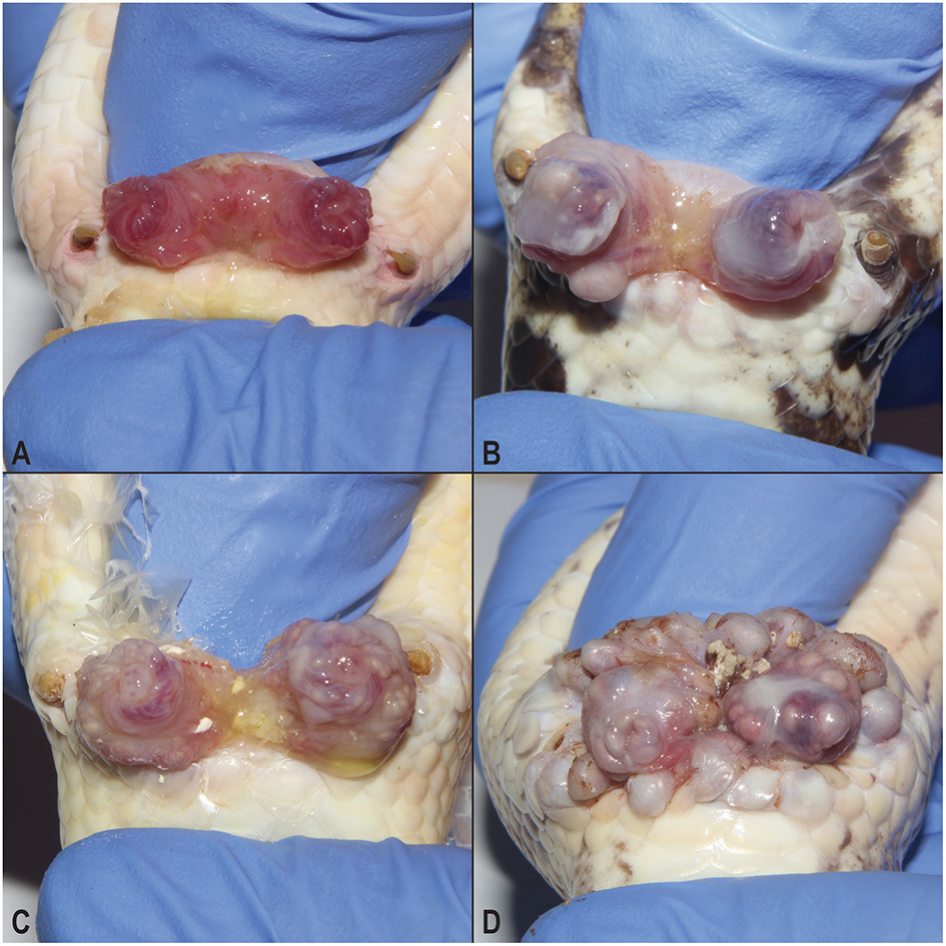
Hemipenal lesions in breeding male ball pythons (*Python regius*). Normal everted ball python hemipenes **(A)** compared to a syndrome of hemipenal inflammation **(B–D)**. Inflammation and irritation ranged from mild **(B)**, to moderate **(C)**, to severe **(D)** which was associated with mass formation along the margins of the vent.

Although irritation on the exterior of cloacas in male ball pythons was noted in the affected facility a decade or more prior to this study, in 2017 an apparent increase in the number of animals with cloacal masses was noted at the facility. Through the practice of manually everting breeding males' hemipenes, a spectrum of expression of the syndrome was observed (Figure 1). In contrast to unaffected snakes (Figure 1A), low (Figure 1B) to moderate (Figure 1C) numbers of 1–3-mm-diameter masses were present within the mucosa of the hemipenes. In the most severely affected individuals, the masses increased in both size (up to 10 mm diameter) and number and were present throughout the mucosa of the hemipenes and surrounding cloacal tissue (Figure 1D).

In the September 2017 cloacal exam census, a gross disease point prevalence of 29.1% was observed in the 481 breeding males in the farm (Table 1), expressing a range of severity in disease expression. Of the 140 affected males, 44 had lesions visible on both the hemipenes and the cloaca, with the remaining 96 males having gross lesions visible on the extruded hemipenes alone. In contrast, only 1 out of 1,446 breeding females presented with the disease syndrome visible on the cloaca. A Chi-square analysis of this data shows sex as being a statistically significant correlation (*P* < 0.00001) with clinical signs of the disease syndrome.

**Table 1:** Epidemiology of gross lesion presence in affected colony from 2017 and 2018 censuses.

**Variable**	**Category**	**Lesions present**	**Lesions absent**	***P***
Year^a^^,^ ^b^^,^ ^c^	2017 breeder male	140 (29.1%)	341 (70.9%)	0.01067^*^
	2018 breeder male	105 (21.9%)	374 (78.1%)	
Sex^a^^,^ ^c^	Breeder male	105 (21.9%)	374 (78.1%)	<0.00001^*^
	Breeder female	1 (<0.1%)	1475 (>99.9%)	
Breeding	Virgin male	0 (0%)	201 (100%)	<0.00001^*^
status^a^^,^ ^c^	Breeding male	105 (21.9%)	374 (78.1%)	
Partner	2017 affected partner	12 (31.6%)	26 (68.4%)	0.00037^*^
status^a^^,^ ^c^	Unaffected partner	40 (10.3%)	348 (89.7%)	

a *Significance determined with Chi-square test, α=0.05*.

b *Data from 2017 census*.

c *Data from 2018 census*.

* *Statistical significance*.

A second census completed by the producer after the completion of the breeding season in October 2018 found a 21.9% gross disease point prevalence in the 479 active breeding males. In contrast, 1 out of 1,477 breeding females had signs of the gross disease syndrome; no virgin males (*n* = 201) or virgin females (*n* = 293) had abnormal gross lesions. Of the 105 males affected with the disease syndrome in the 2018 census, 52 were new cases, consistent with an annual incidence rate of 12.8%; moreover, four of the new cases were of the 166 first-year breeding males. In comparison, none of the 24 virgin males of the same age cohort but not used for breeding in the 2018 season developed any gross signs (*p* = 0.4421). In contrast, only one female developed cloacal lesions in the same timeframe.

In comparison to 21.9% of breeding males (*n* = 479), none of the 201 virgin males showed any expression of disease syndrome, with breeding status correlating significantly (*P* < 0.00001) with the presence of the disease syndrome (Table 1). Of the animals that did not have lesions in 2017 but were placed in a breeding group with a symptomatic male for the 2018 season, 31.6% (*n* = 38) developed lesions in the following year, compared to 10.3% (*n* = 388) of males paired with a partner without apparent lesions; the presence of a breeding partner with clinical signs correlated significantly with the development of signs in the new cases (*P* = < 0.00001; Table 1).

Table 2 shows disease syndrome prevalence and annual incidence compared to age in male ball pythons from the 2018 census. While only 2.41% of animals 2 years in age showed clinical signs in the 2018 census (*n* = 166), animals 3 years in age had a 11.84% prevalence (*n* = 76). Likewise, incidence increases from 2.41% for 2-year-old animals to 5.63% for animals 3 years in age. This positive trend continues for both incidence and prevalence as age increases, ramping up to 66.67% of animals 7 years in age or older expressing the disease syndrome with an annual incidence of 50% (*n* = 57).

**Table 2:** Prevalence and annual incidence of gross lesion presence of males in affected colony from 2018 census.

**Age (Years)**	***n***	**Prevalence%**	**Annual incidence%**
2	166	2.41	2.41
3	76	11.84	5.63
4	73	15.07	10.14
5	60	36.67	15.56
6	47	44.68	29.73
7+	57	66.67	50.00

### Postmortem Examinations

**Figure 2:**
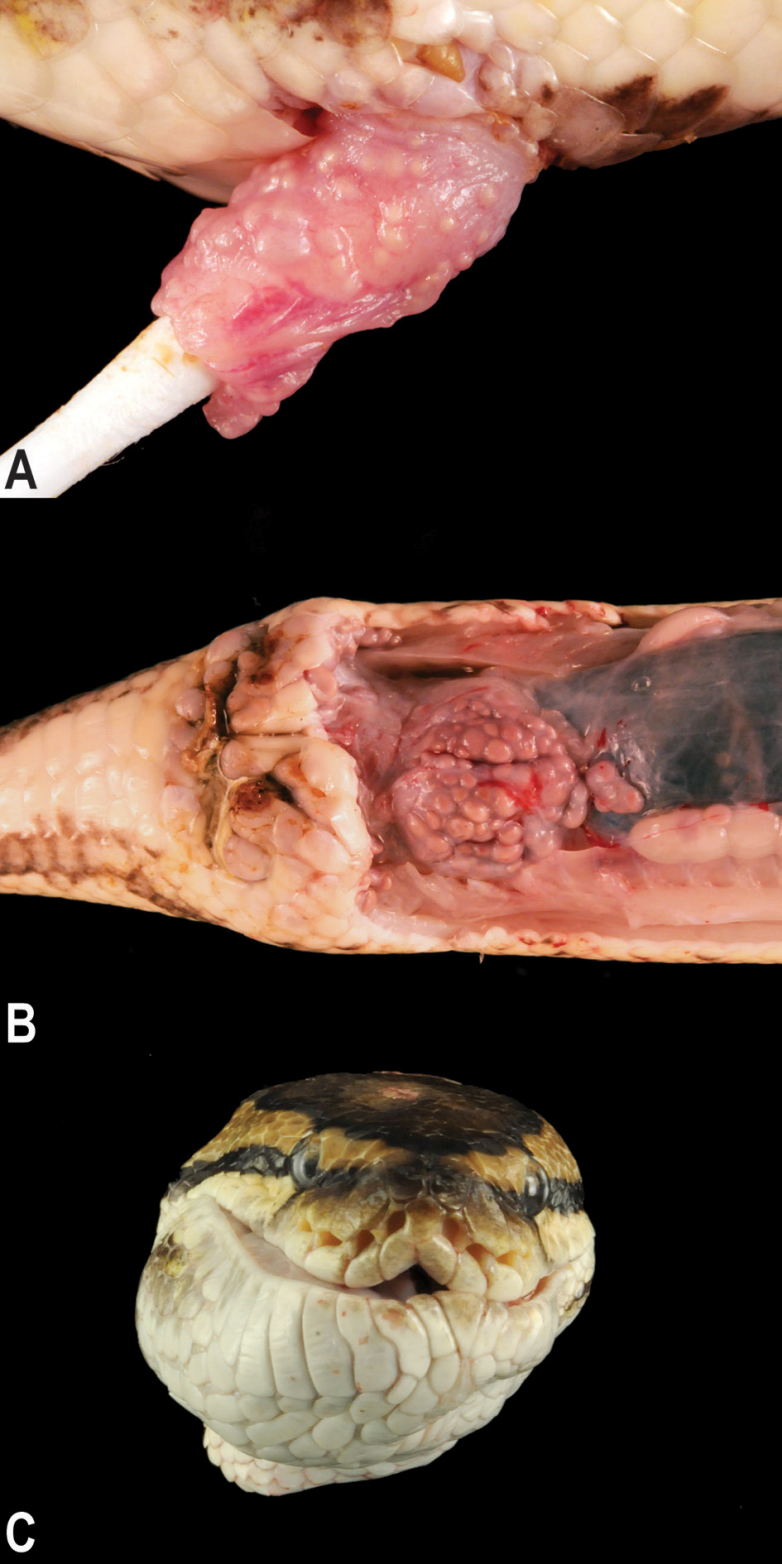
Gross granulomatous inflammation of the hemipenes, cloaca, and oral cavity. Multifocal to coalescing chronic granulomas stud the mucosa of the hemipenis **(A)** are present within the subcutis surrounding the vent and track along the surface of the coelomic cavity deep into the muscle of the ventral body wall **(B)**. Large subcutaneous granulomas deep to the skin and mucosa of the mandible and rostrum distort the profile of the head **(C)**.

Postmortem examinations of the affected snakes revealed multifocal to coalescing, pale tan, smooth, firm, pericloacal, hemipenal, and pericoelomic granulomas. The granulomas were present within the mucosa and submucosa of the hemipenes in males (Figure 2A), expanded the subcutis surrounding the vent, and variably tracked along the surface of the coelomic cavity deep into the muscle of the ventral body wall (Figure 2B). In animals with facial changes, granulomas were present in the submucosa and dermis of the rostrum and mandible that distorted the profile of the head and variably projected into the oral cavity (Figure 2C). Two of the 17 necropsied animals exhibited intracoelomic and/or intrahepatic granulomas of similar appearance.

**Figure 3:**
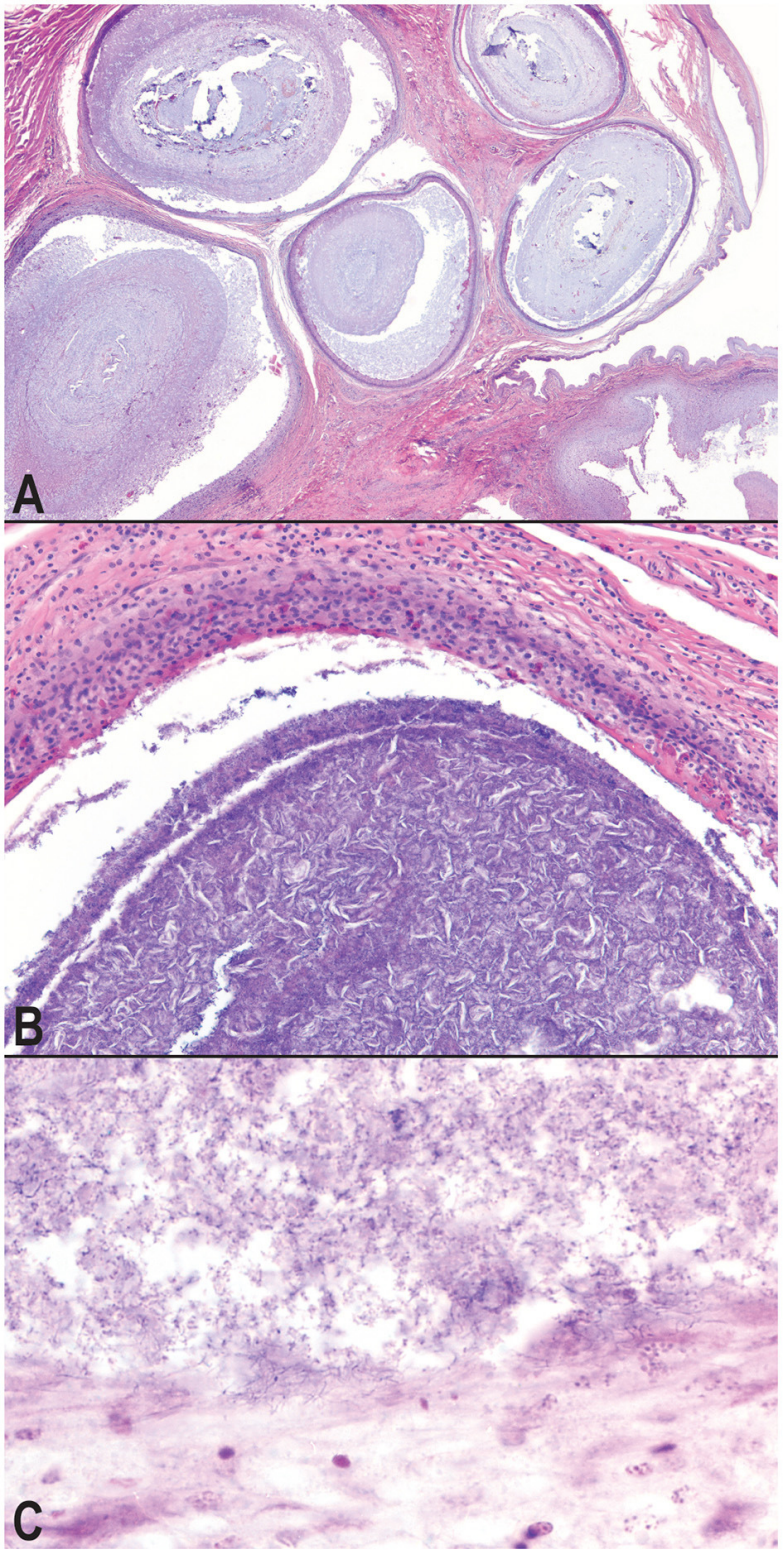
Photomicrographs of granulomatous inflammation of the hemipenes and cloaca. Large, well-demarcated granulomas expand and compress the submucosa and mucosa of the hemipenes; hematoxylin and eosin, 20× magnification **(A)**. Granulomas surround cores of cellular debris, acicular (cholesterol) clefts, mineral, and mixed morphology bacteria; hematoxylin and eosin, 100× magnification **(B)**. A consistent subpopulation of filamentous, Gram-positive bacteria are present throughout the granulomas, and at the highest concentration of the margins of the granulomas with extension into the wall of the granuloma, Gram, 600× magnification **(C)**.

Microscopic examination of the affected tissues identified well-demarcated, chronic granulomas within (i) the mucosa and submucosa of the hemipenes (Figure 3A); (ii) the mucosa, submucosa, dermis, and skeletal muscle surrounding the cloaca; (iii) within the serosa and mesentery of the intestines; (iv) within the oral mucosa, submucosa, dermis, and skeletal muscle of the mandible and rostrum; and (v) occasionally within the parenchyma of the liver and kidney. The granulomas were characterized by a central core of necrotic debris with basophilic, finely granular material (mineral) admixed with moderate to high numbers of clear, acicular spaces (cholesterol clefts; Figure 3B). Necrotic cores were surrounded by a collar of epithelioid macrophages that were often infiltrated by low to moderate numbers of lymphocytes and heterophils. Within granulomas, low to moderate numbers of mixed bacteria were often identified; most prominently present at the margins of the granulomas and extending through the granuloma capsule was a consistent subpopulation of fine, filamentous, Gram-positive bacterial rods, consistent with an actinomycete (Figure 3C). The cloacal granulomas of the lone grossly affected female snake, as well as the two snakes with the hepatic granulomas, and all of the snakes (*n* = 4) with oral granulomas were histologically identical to the granulomas present in the cloaca and hemipenes of the male snakes.

### Bacterial Culture, 16S rRNA PCR, and Phylogenetic Analysis

Successful bacterial growth of the cultured hepatic granuloma was observed on BAP plates; colonies were pinpoint, white, pitting, and adhered to the media. Gram stain showed filamentous Gram-positive bacilli. PCR amplification and Sanger sequencing of the 16S small subunit ribosomal RNA gene resulted in a 1470-nt fragment after editing. A BLASTn search found that the sample most closely matched with members of *Actinomycetaceae* with the closest isolate being *Actinomyces hordeovulneris* (Genbank KF030211.1) with 91.7% nucleotide identity. The sequence was uploaded to GenBank (MT742158).

**Figure 4:**
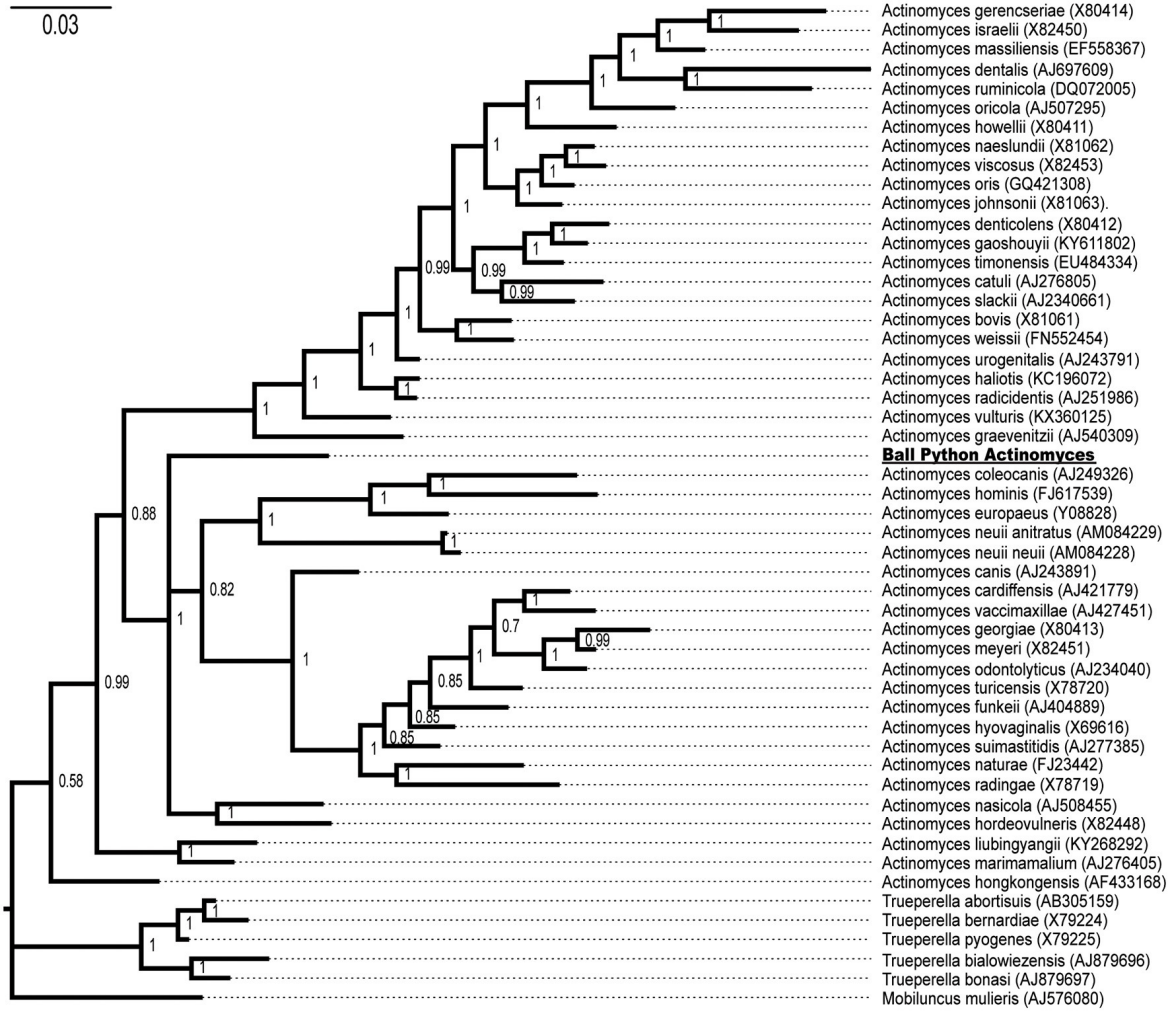
Phylogenetic tree of the isolated ball python bacterium with members of the genera *Actinomyces, Trueperella*, and *Mobiluncus*. Genbank accession numbers are shown in parentheses at the end of the species name. Posterior probabilities are shown at branch points. The ball python *Actinomyces* species is underlined.

Both Bayesian (Figure 4) and maximum likelihood (data not shown) methods of phylogenetic inference placed the novel ball python bacterium within the genus *Actinomyces* with a Bayesian posterior probability of 100% (Figure 4) and a ML bootstrap value of 100% (data not shown), consistent with the isolated snake bacterium as a novel member of the genus *Actinomyces*. This *Actinomyces* will hereafter be referred to as Ball Python *Actinomyces* (BPA).

### Specific BPA Heminested PCR of Tissue and Swab Samples

The BPA 16S rRNA heminested PCR resulted in an amplicon of 420 nucleotides after primer editing. All sequence data matched the homologous region of the sequence amplified from the isolated colony.

BPA was detected by PCR in cloacal/hemipenal granulomas of all 13 necropsied pythons with cloacal and hemipenal lesions. BPA was also detected in cloacal/hemipenal swabs taken from 10/12 (83.3%) snakes with cloacal/hemipenal lesions at necropsy. In the four snakes necropsied with oral/mandibular/facial granulomas, BPA was present in oral tissue in three (75%) of the snakes. BPA was further detected in oral swabs in three of the four (75%) snakes.

Despite a lack of the disease syndrome in the unaffected colonies, cloacal BPA prevalence between the unaffected and affected colonies appear quite similar. The PCR prevalence of BPA was 30.8% (*n* = 65) compared to 42.9% (*n* = 42) in the unaffected colonies. Breeder males within the unaffected colonies had a BPA prevalence of 90% (*n* = 10), compared to 83.3% (*n* = 12) of breeder males with the disease syndrome from the affected colony. Breeder females within the unaffected colonies had a BPA prevalence of 67.7% (*n* = 12), compared to 45.4% (*n* = 11) of breeder females exposed to males with the disease syndrome from the affected colony. Virgin animals in both unaffected and the affected colonies had a markedly lower prevalence of BPA, with 5% (*n* = 20) in unaffected colonies, and 11.9% in the affected colony (*n* = 42).

Swab BPA results from the affected colony are presented in Table 3. Within the affected colony, sex of the animal was not significantly correlated with (*p* = 0.812) the prevalence of BPA, being present in the cloaca of 28.6% of males (*n* = 42) and 34.8% of females (*n* = 23). In contrast, breeding status was significantly correlated (*p* < 0.00001) with the PCR prevalence of BPA, with 65.5% of breeding animals (*n* = 23) having the bacterium compared to 11.9% of virgin animals (*n* = 42). To determine the level of agreement between PCR results and lesion presence in the affected colony, an unweighted Cohen's Kappa test gave a value of 0.56 (*n* = 65, 95% CI [0.336, 0.784]). When expanded to include data from the 2 unaffected colonies, an unweighted Cohen's Kappa test gave a value of 0.138 [*n* = 107, 95% CI (0.138, 0.473)]. This signifies a weak agreement between the presence of BPA by PCR in the cloaca and presence of cloacal lesions.

**Table 3:** Epidemiology of BPA PCR status via swab in affected colony.

**Variable**	**Category**	**Cloaca PCR positive**	**95% CI**	***P***
Lesion presence^a^	Lesions	11/13 (84.6%)	54.6–98.1%	<0.00001^*^
	No lesions	9/52 (17.3%)	8.2–30.3%	
Sex^a^	Male	12/42 (28.6%)	15.7–44.6%	0.812
	Female	8/23 (34.8%)	16.4–57.3%	
Breeding status^a^	Virgin	5/42 (11.9%)	4.0–25.6%	<0.00001^*^
	Breeder	15/23 (65.5%)	42.7–83.6%	
Cloacal PCR vs.	Oral PCR Pos	2/3 (66.7%)	9.4–99.2%	0.2407
oral PCR^b^	Oral PCR Neg	10/34 (29.4%)	15.1–47.5%	

a *Significance determined with Chi-square test, α = 0.05*.

b *Fisher's exact test, α = 0.05*.

* *Statistical significance*.

In the final September 2019 oral examination census, 6 out of 2,027 breeding snakes (2.96%) had oral swelling, all of which were breeder females. PCR testing for BPA in oral swabs of 48 randomly selected animals (12 virgin males, 12 virgin females, 12 breeder males, and 12 breeder females) yielded only 2 snakes (4.2%) testing positive. Oral swabs taken from an additional 37 snakes (17 breeder females, 4 breeder males, 8 virgin females, and 8 virgin males) with prior testing for BPA in the cloaca yielded 5.4% being positive for BPA on both oral and cloacal swabs, 2.7% positive on just the oral swab, 27.0% positive on just the cloacal swab, and 64.9% negative on both swabs (*n* = 37). Fisher's exact test failed to find statistical significance between presence of BPA in the cloaca and presence of BPA in the oral cavity (*p* = 0.2407) (Table 3).

## Discussion

This study characterizes a granulomatous disease syndrome of the cloaca, external reproductive organs, and oral cavity of captive ball pythons (*Python regius*) associated with a novel *Actinomyces* species. The most interesting facet of the disease syndrome is the high proclivity in affecting the external genitals and cloaca of adult breeding male snakes. The histologic features of the lesion, the presence of intralesional, filamentous, Gram-positive bacteria, the aerobic bacterial culture of an actinomycete from affected tissues, and the detection of the cultured bacterium by PCR in affected tissues all support a role of the novel actinomycete in the observed disease syndrome.

*Actinomyces* are common inhabitants of the oral and genital mucosa in both human and animal species. Although often innocuous members of the resident microbiome, a disruption of the mucosal barrier can lead to clinical *Actinomyces* infections (7, 14). In animals, lumpy jaw is a well-characterized disease syndrome that can be caused by several species of *Actinomyces*; the disease is most commonly observed in ruminants, but it can also occur in other mammals including humans (10, 22–25). The lesions of lumpy jaw include facial inflammation and abscess formation with deterioration of underlying bone structures in the oral cavity and jaw. In humans, actinomycetes are known causative agents of pelvic inflammatory disease in females with intrauterine birth control devices (IUDs) (7, 26). Actinomyces infections are not limited to the oral cavity or reproductive tracts, however, and infections of the abdomen, neck, pleura, lungs, liver, kidney, appendix, caecum, skin, heart, meninges, and bone have all been reported (7). In all of these infections, a common characteristic is granulomatous inflammation often with a capsule of connective tissue and swelling (7, 10, 24). Both the observed cloacal/hemipenal and facial lesions of the snakes in this report exhibit features consistent with actinomycosis in other species.

Another facet in the pathogenesis of actinomycosis is the high propensity for polymicrobial infections (22, 27–30). 95.5% of cases of human cervicofacial actinomycoses (*n* = 1997) were polymicrobial mixed infections (22). This is consistent with the histologic appearance of mixed morphology bacteria in the granulomas present in necropsied animals presented here. Moreover, initial attempts to culture a cloacal granuloma from one of the initial presenting snakes resulted in mixed Gram-negative and Gram-positive growth (data not shown). The companion bacteria in polymicrobial actinomycosis are thought to play a synergistic role in the development of actinomycosis (7, 14, 29), possibly by promoting infection through inhibiting host defense and optimizing ideal aerobic conditions (31). Actinomyces infections also commonly spread to adjacent tissue (27, 28, 32) with a subset of cases also presenting with hepatic infection (14, 32, 33). Both these attributes are consistent with what was observed in necropsied animals. While only a small subset (*n* = 2) of snakes had hepatic granulomas, regional extension of granulomas was a very common finding in the snakes (see Figure 2B).

While this is the first documentation of an *Actinomyces* species as a cause of granulomatous cloacal/hemipenal disease in snakes, this is not the first report of such lesions in a ball python. A figure in a review of snake reproduction by Stahl (4) showed similar lesions in the hemipenes of a male ball python. The biopsy was reported to reveal acid-fast positive bacterium; while this result was interpreted as mycobacteriosis, some *Actinomyces* spp. have been reported to exhibit acid-fast reactivity (34). Moreover, in addition to the large colony described in this paper, anecdotal observation of the disease syndrome has been reported in two other large (1000+ animal) colonies and two smaller (300+ animal) colonies (personal communication, Tillis). Lastly, a recent diagnostic biopsy submission to the Aquatic, Amphibian, and Reptile Pathology service at the University of Florida from a ball python was composed of cloacal granulomas histologically identical to those identified in this population and contained filamentous, Gram-positive bacilli (personal communication, Ossiboff). Together, these suggest that actinomycosis may not just be a sporadic event in this one breeding colony of ball pythons, and clinicians and hobbyists should be aware of this disease condition.

In the colony described here, the uptick in the disease syndrome that prompted this investigation was correlated with an increase in colony size of 22% from the year prior. Combined with the observations of a similar disease in other large collections, colony size may play a role in the prevalence of the disease syndrome. However, studies of disease transmission in livestock animal production have shown disease transmission rates to be independent of population size (35–38). Host density may have a larger role, and increase in population size without corresponding space increase may affect transmission rates (39). Farm-level surveys would be required to identify farm-level risk factors for the disease syndrome.

While both oral and cloacal/hemipenal lesions were observed in snakes in this colony, oral masses and PCR detection of BPA in oral swabs occurred at a markedly lower rate than that in the cloaca. This finding may suggest that the cloaca is either a more hospitable environment for BPA to grow, or frequent direct cloacal contact during breeding is a more efficient means of bacterial transmission within the colony. Although the environmental persistence of BPA and its role in transmission dynamics of the disease syndrome is unknown, presence of the disease syndrome was only seen in snakes with a history of breeding activity. Lack of the disease syndrome in virgin animals as well as the statistically significant increase in the development of the disease syndrome in unaffected males partnered with affected males could indicate venereal spread of the disease syndrome. An increased BPA prevalence was also associated with active breeding status animals. Due to the qualitative nature of the developed PCR test, BPA could only be confirmed as present or absent in the cloaca. Without a quantitative assay, relative BPA levels in the cloaca of PCR-positive animals with the disease syndrome cannot be compared to bacterial counts of BPA PCR positive animals without the disease syndrome.

The reproductive sequelae of the disease syndrome for affected snakes are unclear. Because this colony has 2 designated males per breeding group, fecundity can only be examined on the breeding group level. A decrease in fecundity brought on by the disease could be missed if the second male in the breeding group masked a deficit in fecundity in the partnering male or if fecundity is only affected in later-stage disease expression. Likewise, the culling of affected animals within the colony may mask late-stage disease progression that could ultimately result in higher untimely mortality. However, the clinical significance for the snakes in advanced stages of disease is unquestionable. Animals with severe disease exhibit other sequela to the disease, including mucosal and cutaneous ulceration, secondary infections, and possible obstipation.

Despite evidence for association with the disease syndrome, the prevalence of BPA did not differ greatly between the affected and unaffected colonies sampled in this study. This could suggest a multifactorial nature of the disease syndrome. One possible explanation for the formation of the disease syndrome presented here is that strenuous, repetitive, and long-term breeding of males at large production facilities can result in hemipenal mucosal damage. This mucosal damage paired with the presence of BPA could then lead to a higher chance of development of actinomycosis, as is well-documented in other species. This explanation would support males presenting with the disease syndrome at far higher rates than females, as well as why no virgin animals experienced the disease syndrome. Furthermore, the presence of a yet unknown co-infective agent, as is also common in actinomycoses in other species, could explain why BPA was associated with the disease syndrome in the affected colony but not in unaffected colonies. However, experimental infections and further studies of pathogenesis would be necessary to fulfill Koch's postulates and show a causative relationship between BPA and the disease syndrome.

## Data Availability Statement

The datasets presented in this study can be found in online repositories. The names of the repository/repositories and accession number(s) can be found in the article/Supplementary Material.

## Ethics Statement

Ethical review and approval was not required for the animal study because all prospective analysis/research was performed for diagnostic purposes for the health of the study collections at the request of the collection owners. All diagnostic testing is covered under University of Florida IACUC protocol #201907944. Written informed consent was obtained from the owners for the participation of their animals in this study.

## Author Contributions

ST, MI, EG, and RO contributed to postmortem examination and interpretation and photographs. ST, AC, JW, and RO contributed to molecular characterization. ST and RI contributed to statistical analysis. All authors contributed to overall study design and writing of the manuscript and approve of the submitted version.

## Conflict of Interest

The authors declare that the research was conducted in the absence of any commercial or financial relationships that could be construed as a potential conflict of interest.
